# Constipation Caused by Anti-calcitonin Gene-Related Peptide Migraine Therapeutics Explained by Antagonism of Calcitonin Gene-Related Peptide’s Motor-Stimulating and Prosecretory Function in the Intestine

**DOI:** 10.3389/fphys.2021.820006

**Published:** 2022-01-11

**Authors:** Peter Holzer, Ulrike Holzer-Petsche

**Affiliations:** Division of Pharmacology, Otto Loewi Research Centre, Medical University of Graz, Graz, Austria

**Keywords:** calcitonin gene-related peptide (CGRP), CGRP receptor antagonists (gepants), CGRP antibodies, CGRP receptor antibodies, migraine, constipation, peristaltic motor activity, diarrhea

## Abstract

The development of small-molecule calcitonin gene-related peptide (CGRP) receptor antagonists (gepants) and of monoclonal antibodies targeting the CGRP system has been a major advance in the management of migraine. In the randomized controlled trials before regulatory approval, the safety of these anti-CGRP migraine therapeutics was considered favorable and to stay within the expected profile. Post-approval real-world surveys reveal, however, constipation to be a major adverse event which may affect more than 50% of patients treated with erenumab (an antibody targeting the CGRP receptor), fremanezumab or galcanezumab (antibodies targeting CGRP). In this review article we address the question whether constipation caused by inhibition of CGRP signaling can be mechanistically deduced from the known pharmacological actions and pathophysiological implications of CGRP in the digestive tract. CGRP in the gut is expressed by two distinct neuronal populations: extrinsic primary afferent nerve fibers and distinct neurons of the intrinsic enteric nervous system. In particular, CGRP is a major messenger of enteric sensory neurons which in response to mucosal stimulation activate both ascending excitatory and descending inhibitory neuronal pathways that enable propulsive (peristaltic) motor activity to take place. In addition, CGRP is able to stimulate ion and water secretion into the intestinal lumen. The motor-stimulating and prosecretory actions of CGRP combine in accelerating intestinal transit, an activity profile that has been confirmed by the ability of CGRP to induce diarrhea in mice, dogs and humans. We therefore conclude that the constipation elicited by antibodies targeting CGRP or its receptor results from interference with the physiological function of CGRP in the small and large intestine in which it contributes to the maintenance of peristaltic motor activity, ion and water secretion and intestinal transit.

## Introduction

Calcitonin gene-related peptide was overtaking many a neuropeptide in its path to clinical exploitation, given that only some 20 years after its discovery (Rosenfeld et al., 1983) a landmark paper (Olesen et al., 2004) provided clinical proof of the concept that a small molecule antagonist of CGRP (code name BIBN4096BS, later given the international non-proprietary name olcegepant) is able to prevent and treat migraine. Since then several small molecule antagonists (as a group termed *gepants*) have been developed for use in migraine management, followed by the successful introduction of monoclonal antibodies targeting CGRP or its receptor (for reviews see Edvinsson, 2019; Hargreaves and Olesen, 2019; Omaer et al., 2021). The results of the randomized controlled trials (RCTs) in phase 3 of clinical development attributed not only satisfactory efficacy but also acceptable safety and tolerability to these CGRP-directed migraine therapeutics. The profile of treatment-emergent adverse effects did not expose any major concern with those compounds that were advanced to regulatory approval. Unexpectedly, though, post-approval real-world surveys revealed constipation to be a major adverse effect of the monoclonal antibodies targeting the CGRP system.

Constipation caused by CGRP antagonism/neutralization is the prime focus of this review article which seeks to provide a mechanistic explanation of this adverse effect which in hindsight was not totally unforeseen. In addressing this issue, we first survey the gastrointestinal adverse effect profile of gepants and monoclonal antibodies targeting either CGRP or the CGRP receptor, collectively termed anti-CGRP migraine therapeutics, that came to light in the phase 3 trials prior to marketing authorization. We then summarize the available data showing constipation to be associated with the use of antibodies targeting CGRP or its receptor in the post-approval stage. Considering the post-approval data we go on to examine the question whether constipation caused by inhibition of CGRP signaling can mechanistically be deduced from the known actions and pathophysiological implications of CGRP in the gastrointestinal tract of laboratory animals and humans. Although we notice that the effects of CGRP in the human gut have insufficiently been studied, we are able to gather plausible evidence that CGRP can stimulate propulsive motility and secretion in the human intestine. If the pharmacological actions of CGRP in the human intestine were to reflect the physiological implications of the peptide, antagonism of CGRP signaling would be expected to cause constipation as shown by the adverse effect of anti-CGRP therapeutics. In addition, we take into account that certain neuroendocrine tumors expressing and releasing CGRP are known to cause watery diarrhea, which indirectly ascribes CGRP a role in promoting propulsive motility in the human intestine. With these considerations in mind we provide a rational explanation why long-term blockade of CGRP signaling results in constipation as a major adverse effect of monoclonal antibodies targeting CGRP or its receptor.

## Gastrointestinal Side Effect Profile of Anti-Calcitonin Gene-Related Peptide Migraine Therapeutics in Pre-Approval Clinical Trials

Although they were efficacious in migraine management, the first gepants including olcegepant (code name BIBN4096BS), MK-3207 and telcagepant (code name MK-0974) did not make it to approval by regulatory authorities for various reasons. One issue was that MK-3207 and telcagepant were liable to enhance serum levels of hepatic aminotransferases and carry a risk of hepatotoxicity (Ho et al., 2008). Concerns about drug-induced liver injury also accompanied the development of further gepants. However, ubrogepant, rimegepant and atogepant have meanwhile been approved as migraine therapeutics by the US Food and Drug Administration (FDA), and other regulatory authorities including the European Medicines Agency (EMA) were following suit in licensing these gepants. The phase 3 trials of these small molecule CGRP receptor antagonists revealed only rare and transient elevations of serum aminotransferase levels (Croop et al., 2019; Dodick et al., 2019; Lipton et al., 2019a,b; Ailani et al., 2020; Omaer et al., 2021) that did not lead to clinically manifest liver injury.

The gepants in clinical use are in general well tolerated as proved in the RCTs during clinical development. Apart from infections of the upper respiratory, urinary and/or gastrointestinal tract, nausea (1.8–4.7% of patients on gepants) was the most common gastrointestinal complaint reported in phase 3 trials of ubrogepant, rimegepant and atogepant (Croop et al., 2019; Lipton et al., 2019a,b; Ailani et al., 2020, 2021; Omaer et al., 2021). Diarrhea was noted in 2.5–2.7% of patients on ubrogepant (Ailani et al., 2020) while constipation was reported in 6.9–7.7% of subjects taking atogepant (Ailani et al., 2021), but were not noticed in the trials of the other gepants. The proven efficacy of gepants in migraine prevention and treatment provided ample evidence for an implication of CGRP in the pathophysiology of migraine and fostered efforts to explore other routes to target the CGRP system.

To avoid the potential hepatic toxicity of some of the gepants, monoclonal antibodies targeting the CGRP system were envisaged as an alternative therapeutic approach. Meanwhile, a number of monoclonal antibodies targeting either the CGRP receptor (erenumab) or CGRP itself (fremanezumab, galcanezumab and eptinezumab) have entered the therapeutic arena. The safety of these monoclonal antibodies during clinical development has been regarded as favorable and to stay within a profile expected for this class of therapeutics (production of anti-drug antibodies, local injection responses, hypersensitivity reactions, infections of the upper respiratory tract). The results of the phase 3 trials also showed that their gastrointestinal adverse effect profile posed no particular concern, although some distinct differences between the antibodies targeting the CGRP receptor and those targeting CGRP emerged.

Fremanezumab appeared to be largely devoid of adverse effects on the gut as the frequency of nausea observed in the antibody-treated patients did not exceed that noted in placebo-treated subjects (Silberstein et al., 2017; Dodick et al., 2018b; Ferrari et al., 2019). No mention of constipation was made in three phase 3 trials (Silberstein et al., 2017; Dodick et al., 2018b; Sakai et al., 2021), whereas one phase 3 study reported constipation occurring in 3% of subjects receiving fremanezumab (675 mg) once every 3 months but not in those receiving fremanezumab (225 mg) monthly (Ferrari et al., 2019). Similarly, constipation and other gastrointestinal adverse effects in individuals on galcanezumab were either not reported or their rate did not exceed that seen in placebo-treated patients (Detke et al., 2018; Skljarevski et al., 2018; Stauffer et al., 2018; Mulleners et al., 2020). Only one phase 3 trial showed nausea to occur in 6.4–7.8% of patients on galcanezumab (Camporeale et al., 2018). Constipation was likewise found to be absent or negligible in five phase 3 studies of eptinezumab (Ashina M. et al., 2020; Lipton et al., 2020; Silberstein et al., 2020; Kudrow et al., 2021; Winner et al., 2021) while low rates of eptinezumab-associated nausea were noted in two phase 3 trials (Lipton et al., 2020; Silberstein et al., 2020).

Unlike the antibodies targeting CGRP (fremanezumab, galcanezumab, eptinezumab), erenumab, an antibody targeting the CGRP receptor, emerged to have some potential to cause constipation in the pre-approval RCTs. Thus, constipation in 1.6–5.1% of patients on erenumab was reported in three phase 3 trials (Goadsby et al., 2017; Tepper et al., 2017; Takeshima et al., 2021) whereas in five other phase 3 trials (Sun et al., 2016; Dodick et al., 2018a; Reuter et al., 2018; Goadsby et al., 2020; Hirata et al., 2021) no mention of constipation was made. Occasional nausea and diarrhea were noted in one phase 3 trial (Goadsby et al., 2020). Taken together, the intestinal adverse effect profile of erenumab was considered to be without substantial concern based on the data collected in the pre-approval trials of the antibody. It is worth mentioning, though, that a constipation rate of 11% was observed when erenumab, but not galcanezumab, was co-administered with ubrogepant in an open-label phase 1b trial of migraine patients (Jakate et al., 2021).

## Constipation as an Adverse Effect of Calcitonin Gene-Related Peptide and Calcitonin Gene-Related Peptide Receptor Antibodies in Post-Approval Surveys

As the development of anti-CGRP migraine therapeutics gained momentum, concerns were raised that blockade of CGRP signaling may have adverse effects, especially if used for long term periods that were not covered by phase 3 trials. In view of CGRP’s vasodilator action (for a review see Russell et al., 2014) it was, for instance, argued that comorbidities in the cardiovascular system may enhance the risk of cerebral and cardiac ischemia (MaassenVanDenBrink et al., 2016; Deen et al., 2017). Since CGRP also plays a role in safeguarding gastrointestinal mucosal blood flow in the face of pending injury (Holzer, 1998; Akiba et al., 2002), antagonism of CGRP’s vasodilator action was regarded to potentially compromise gastrointestinal mucosal homeostasis. Indeed, neutralization of CGRP with an antibody or CGRP knockout prevents the protective effect of capsaicin, a stimulant of extrinsic sensory neurons releasing CGRP, against ethanol-induced damage in the rat gastric mucosa (Peskar et al., 1993; Ohno et al., 2008). Likewise, experimental colitis evoked by trinitrobenzene sulfonic acid in rodents is aggravated by the CGRP receptor antagonist CGRP_8–37_, a CGRP antibody (Reinshagen et al., 1998), and genetic deletion of CGRP (Engel et al., 2011). CGRP antagonism may thus interfere with the protective function of CGRP released from sensory nerve fibers in the gastrointestinal mucosa (Holzer, 1998; Akiba et al., 2002), a function that may include facilitated wound healing and neovascularization (Ohno et al., 2008). Fortunately, this adverse event potential of anti-CGRP therapeutics in the gastrointestinal mucosa has thus far not been substantiated in the RCTs and post-approval surveys.

The concerns raised by Deen et al. (2017) and Hargreaves and Olesen (2019) that CGRP antagonism/neutralization may bear a risk of constipation, though, has been corroborated to a remarkable degree in post-approval studies and surveys. The predictive validity of this concern was uncertain at that time, since any long-term effects of CGRP-targeting migraine therapy were not yet known and the pathophysiological implications of CGRP in humans remained largely unexplored. In addition, the ongoing phase 3 trials did not disclose any substantial gastrointestinal adverse effect potential of the anti-CGRP migraine therapeutics under clinical development. It therefore came as an unforeseen surprise that in post-approval real-world surveys constipation emerged as a major unwanted side effect of monoclonal antibodies targeting the CGRP system. Table 1 summarizes the pertaining reports that have been published to date and shows that the available real-world evidence for constipation relates, at the present time, to the monoclonal antibodies targeting the CGRP system.

**TABLE 1:** Constipation rates in migraine patients treated with CGRP receptor antagonists or monoclonal antibodies targeting CGRP or its receptor as reported in post-approval surveys (real-world evidence).

Compound	Target	Recommended dosing	Treatment duration	Constipation rate	References
Ubrogepant	Small molecule CGRP receptor antagonist	50 or 100 mg p.o. twice daily	1–3 Months after prescription (50 or 100 mg)	4.7%	Chiang et al., 2021
Erenumab	Monoclonal anti-CGRP receptor antibody	70 or 140 mg s.c. once a month	6-month follow-up (70 mg optionally followed by 140 mg after 3 months)	65%	de Vries Lentsch et al., 2021
			8-month survey period (70 or 140 mg)	43%	Kanaan et al., 2020
			3-month survey period (140 mg)	34%	Ashina H. et al., 2020
			6-month follow-up (70 or 140 mg)	20% (70 mg erenumab), 32.6% (140 mg erenumab)	Alex et al., 2020
			6-month follow-up (70 mg optionally followed by 140 mg after 3 months)	23.9%	Russo et al., 2020
			3-month treatment (70 mg)	23.8%	Scheffler et al., 2020
			6-month survey period (70 or 140 mg)	23.6%	Robblee et al., 2020
			16-month survey period (data combined for erenumab, galcanezumab and fremanezumab, doses not specified)	23%	Robbins and Phenicie, 2020
			13-month survey period (at least 3 months on erenumab, 70 or 140 mg)	21%	Faust et al., 2021
			3-month treatment (data combined for erenumab, 70 mg, galcanezumab and fremanezumab)	21%	Scheffler et al., 2021
			Duration and specific dose not specified	20%	Robbins, 2019
			6-month follow-up (70 mg, with the option to increase dose to 140 mg in months 4–6)	20% at month 1 11% at month 3 5% at month 6	Lambru et al., 2020
			3-month survey period (data combined for erenumab, 140 mg, and galcanezumab, 120 mg, after loading dose of 240 mg)	20%	Torres-Ferrús et al., 2021
			6-month follow-up (70 mg optionally followed by 140 mg after 3 months)	20%	Silvestro et al., 2021
			12-month survey period (70 or 140 mg)	16.7%	Eghtesadi et al., 2021
			6-month survey period (70 mg optionally followed by 140 mg after 3 months)	13.5%	Ornello et al., 2020
			18-month survey period (70 or 140 mg)	7.6%	Belvís et al., 2021
Galcanezumab	Monoclonal anti-CGRP antibody	120 mg s.c. once a month, with a first loading dose of 240 mg s.c.	16-month survey period (data combined for erenumab, galcanezumab and fremanezumab, doses not specified)	23%	Robbins and Phenicie, 2020
			3-month survey period (data combined for erenumab, 140 mg, and galcanezumab, 120 mg, after loading dose of 240 mg)	20%	Torres-Ferrús et al., 2021
			6-month follow-up (240 mg loading dose followed by 120 mg once a month)	17.4%	Alex et al., 2020
Fremanezumab	Monoclonal anti-CGRP antibody	225 mg s.c. once a month or 675 mg s.c. every 3 months	6-month follow-up (225 mg once a month)	25%	Alex et al., 2020
			16-month survey period (data combined for erenumab, galcanezumab and fremanezumab, doses not specified)	23%	Robbins and Phenicie, 2020

The data put together in Table 1 also demonstrate that constipation is a troubling adverse effect independently of whether the antibodies neutralize the CGRP receptor or CGRP itself. As the surveys show, constipation may affect more than 50% of patients receiving erenumab. It is worth noting that the lowest rate of erenumab-associated constipation (7.6%) was found in Spanish patients (Belvís et al., 2021), while the highest rates (32.6–65%) were found in Dutch (de Vries Lentsch et al., 2021) and US (Alex et al., 2020; Kanaan et al., 2020) patients as well as in a Danish cohort of patients with headache attributed to mild traumatic brain injury (Ashina H. et al., 2020). A more detailed analysis indicates that constipation due to erenumab depends on dose (Alex et al., 2020) and is most prominent at the start of treatment (Lambru et al., 2020). Antibodies targeting CGRP itself appear to be equally liable to cause constipation as erenumab (Table 1). Thus, constipation rates of 17.4–23% were reported for galcanezumab and rates of 23–25% for fremanezumab (Alex et al., 2020; Robbins and Phenicie, 2020; Torres-Ferrús et al., 2021).

As regards the small molecule CGRP receptor antagonists, only one post-approval survey has been published, showing that 4.7% of patients on ubrogepant complained about constipation (Chiang et al., 2021). As described in the previous section of this article, no mention of constipation as an adverse effect was made in the phase 3 trials of ubrogepant. Whether the real world constipation rates for other gepants will also be higher than those in the pre-approval RCTs remains to be examined. Such a post-approval evaluation is in particular awaited for atogepant which in one phase 3 trial was reported to cause constipation in 6.9–7.7% of patients (Ailani et al., 2021).

The pre- and post-approval studies/surveys considered here were selected in order to provide valid evidence for constipation being an unwanted action of CGRP migraine therapeutics and to underline the need to explain this unforeseen adverse effect. There are, however, some limitations inherent in these studies. Firstly, gepants are indicated for the acute migraine treatment while antibodies targeting the CGRP system are used for preventive migraine management. Secondly, the post-approval studies/surveys of CGRP-directed antibodies have typically been conducted in migraine patients that had a history of multiple treatment failures. Thirdly, in many studies the concomitant use of one or more preventive headache medications was permitted. If the migraine patients continue treatment by a polypharmacy approach including, for instance, opioid receptor agonists, the rate of constipation may be equally high before and after erenumab therapy (Faust et al., 2021).

The high rates of constipation disclosed by real world evaluation of the efficacy and safety of antibodies targeting the CGRP receptor or CGRP itself emphasize the need to closely monitor their intestinal adverse effect profile and in this way safeguard the success in migraine treatment. In this context it is also important to address the question in which way self-rated constipation can be classified in a clinically meaningful manner. Assessment of drug-induced constipation is not a trivial task because it is important to differentiate between self-reported constipation and constipation assessed by objective criteria as, for instance, proposed for opioid-induced constipation (Lacy et al., 2016; Farmer et al., 2019). Self-assessed constipation is dominated by individual perceptions such as ease of defecation or incomplete bowel emptying and individual expectations in the frequency of bowel movements. This may also in part explain why the rates of self-reported constipation vary considerably among the different surveys listed in Table 1. The use of diagnostic procedures for objective assessment of constipation, e.g., the Rome IV criteria (Lacy et al., 2016) or the functional bowel index (Farmer et al., 2019), may yield results appreciably different from self-reported constipation rates. Apart from differences in individual perception and expectation, patients may also overlook gradual changes in bowel movements that could have taken place before a CGRP-targeting therapy was begun. It should not go unnoticed in this context that migraine as well as non-migrainous headache can be comorbid with gastrointestinal disturbances including constipation (Kelman, 2004; Aamodt et al., 2008). Notwithstanding these considerations, the post-approval survey data indicate that a considerable part of migraine patients treated with antibodies against CGRP or the CGRP receptor suffer from constipation severe enough to curtail their quality of life.

## Evidence for a Role of Calcitonin Gene-Related Peptide in Stimulating Intestinal Propulsion and Secretion in Laboratory Animals

### Innervation of the Gut by Calcitonin Gene-Related Peptide-Expressing Neurons

**FIGURE 1:**
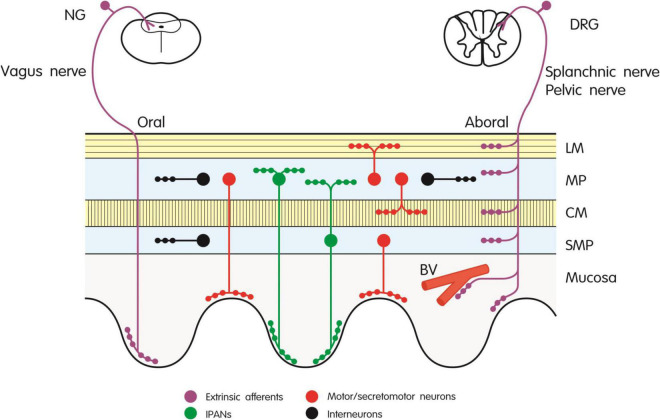
Innervation of the rodent gut by extrinsic afferent and intrinsic enteric neurons expressing CGRP. The different types of neuron are depicted by different color codes as explained in the graph. BV, blood vessel; CM, circular muscle; DRG, dorsal root ganglion; IPANs, intrinsic primary afferent neurons; LM, longitudinal muscle; MP, myenteric plexus; NG, nodose ganglion; SMP, submucosal plexus.

**FIGURE 2:**
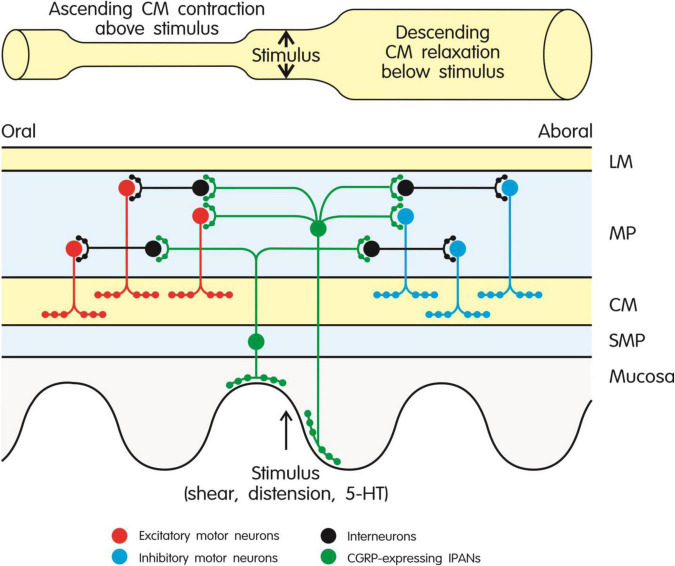
Role of CGRP-expressing intrinsic primary afferent neurons (IPANs) in the peristaltic motor activity of the intestine through activation of ascending excitatory and descending inhibitory pathways in the enteric nerve plexus. CGRP-expressing IPANs are stimulated by mechanical or chemical stimulation of the mucosa and intestinal wall. Ascending contraction of the circular muscle above the stimulus is mediated by interneurons and excitatory cholinergic motor neurons, while descending relaxation of the circular muscle below the stimulus is brought about by interneurons and inhibitory motor neurons (expressing nitric oxide synthase and various relaxant mediators). The different types of neuron are depicted by different color codes as explained in the graph. The model shown in the graph takes particular account of the findings reported by Grider et al. (1998) and Smolilo et al. (2020). CM, circular muscle; 5-HT, 5-hydroxytryptamine; LM, longitudinal muscle; MP, myenteric plexus; SMP, submucosal plexus.

In our attempt to explain the potential of CGRP antagonism/neutralization to induce constipation we first review experimental studies in laboratory animals for relevant effects of CGRP and CGRP antagonism on gastrointestinal motor activity and secretory processes. The major sources of CGRP in the gut of rodents are extrinsic afferent nerve fibers and intrinsic enteric neurons (for early reviews see Taché et al., 1991; Sternini, 1992; Holzer, 1994, 2000; Maggi, 1995), and this dual CGRP innervation holds also true for the digestive tract of dogs and pigs (Sternini et al., 1992; Rasmussen et al., 2001). Figure 1 provides a schematic diagram of the neuronal sources of CGRP in the rodent gut. In the rat, most of the CGRP expressed in extrinsic sensory neurons is α-CGRP, whereas the peptide found in enteric neurons is β-CGRP (Mulderry et al., 1988; Sternini and Anderson, 1992). In the mouse, β-CGRP appears to be the only form of the peptide occurring in the intestine (Schütz et al., 2004). While the major source of CGRP in the foregut of rat, mouse and guinea-pig are extrinsic afferent neurons, intrinsic enteric neurons constitute the major source of the peptide in the small and large intestine (Sternini et al., 1987; Su et al., 1987; Green and Dockray, 1988; Mitsui, 2011). CGRP-immunoreactive neurons of the myenteric and submucosal plexus in the guinea-pig intestine extend abundant processes to the mucosa (Furness et al., 1985). Similarly, CGRP has been localized to cholinergic secretomotor neurons in the submucosal plexus of the mouse ileum (Mongardi Fantaguzzi et al., 2009). In the rat intestine CGRP-expressing neurons have been found to issue oral or caudal projections within the nerve plexus as well as to the muscle layers and the mucosa (Ekblad et al., 1987; Sternini et al., 1987). CGRP is a particular chemical code of intrinsic primary afferent neurons (IPANs) which originate from the enteric nerve plexus of the mouse, rat and guinea-pig intestine (Figure 2) and have direct connections with both excitatory and inhibitory enteric motor pathways (Furness et al., 2004; Mitsui, 2009, 2011; Hibberd et al., 2018; Smolilo et al., 2020). Likewise, CGRP occurs in more than 90% of IPANs (neurons with a Dogiel type II morphology) in the myenteric plexus of the porcine small intestine, but is also expressed in other types of neuron (Brehmer et al., 2002; Wolf et al., 2007).

The CGRP-expressing extrinsic afferent neurons in the rodent gut (Figure 1) originate primarily from cell bodies in the dorsal root ganglia (Sternini et al., 1987; Green and Dockray, 1988; Mulderry et al., 1988; Sternini and Anderson, 1992; Robinson et al., 2004; Tan et al., 2008; Chen et al., 2016). Within the gastrointestinal tract, they supply primarily the arterial system but also project to the submucosal and myenteric nerve plexus, to the circular and longitudinal muscle layers and the lamina propria of the mucosa (Sternini et al., 1987; Su et al., 1987; Green and Dockray, 1988). CGRP-positive vagal afferents originating from the nodose ganglia make a limited contribution to the CGRP content of the rat foregut, supplying primarily the mucosa but not the muscle layers (Sternini and Anderson, 1992; Bäck et al., 1994; Suzuki et al., 1997; Tan et al., 2009).

### Expression of Calcitonin Gene-Related Peptide Receptors in the Gut and Functional Evidence for a Role of Calcitonin Gene-Related Peptide in Stimulating Propulsive Motility

CGRP has been reported to have multiple actions in the gastrointestinal tract, influencing motor, secretory, vascular and immune functions (for a review see Holzer, 2000), these implications depending on the innervation of the target tissues by CGRP-releasing neurons and the expression of CGRP receptors by the effector cells. The localization of specific CGRP binding sites to the enteric nerve plexus and other tissues in the canine and rat digestive tract (Gates et al., 1989; Ozdemir et al., 1999) has been confirmed by the presence of CGRP receptors in the myenteric nerve plexus of the human gastrointestinal tract (Cottrell et al., 2012). The functional implications of CGRP receptors cannot only be studied by the use of CGRP receptor antagonists and antibodies targeting CGRP or the CGRP receptor but also by (genetic) modification of the expression of CGRP receptors. With regard to the elucidation of gastrointestinal CGRP functions, the latter approach has not yet been employed to any significant extent. This may in part be related to the fact that functional CGRP receptors are heterodimers of calcitonin receptor-like receptor (CLR) and receptor activity-modifying protein-1 (RAMP1) (for a review on CGRP receptor pharmacology see Hay et al., 2018). There is still a lack of studies localizing functional CGRP (CLR/RAMP1) receptors to particular effector systems and matching the presence of these receptors with the pharmacological actions of CGRP in the gut.

In explaining the potential of CGRP antagonism/neutralization to cause constipation it is of prime relevance to consider the known pharmacological and physiological roles that CGRP plays in the regulation of gastrointestinal motor activity. Given the multiple sources of CGRP in the gut it is to be expected that the pharmacological actions of CGRP are complex, comprising both excitatory and inhibitory actions on gastrointestinal motility. CGRP causes muscle relaxation in the guinea-pig and rat small and large intestine by a direct action on the muscle (Holzer et al., 1989; Barthó et al., 1991; Barthó and Holzer, 1995; Sun and Benishin, 1995; Maggi et al., 1996; Rekik et al., 1997; Clifton et al., 2007), an effect that is also seen when CGRP is released from extrinsic sensory neurons stimulated by capsaicin (Barthó et al., 1991; Barthó and Holzer, 1995). Defunctionalization of extrinsic CGRP-releasing neurons by capsaicin, however, as well as CGRP antagonism by CGRP_8–37_ do not modify distension-induced peristalsis in the isolated guinea-pig small intestine (Barthó and Holzer, 1995). In contrast, release of CGRP from extrinsic sensory neurons slows gastric emptying in the rat (Forster and Dockray, 1991) as does intravenous or intraperitoneal administration of CGRP to dogs (L’Heureux et al., 2000) and rats (Lenz, 1988; Raybould et al., 1988; Plourde et al., 1993; Julia and Buéno, 1997) through inhibition of gastric corpus motility (Zittel et al., 1994). The inhibitory action of CGRP released from extrinsic sensory neurons on gastrointestinal motility plays an important role in the ileus following abdominal surgery. Capsaicin-induced ablation of extrinsic spinal sensory neurons, CGRP antagonism by CGRP_8–37_ or olcegepant, and CGRP immunoneutralization in the mouse, rat and dog are all able to reverse postoperative ileus in terms of inhibition of gastric corpus motility (Zittel et al., 1994), inhibition of gastric emptying (Plourde et al., 1993; Freeman et al., 1999; Trudel et al., 2003) and inhibition of transit through the small and large intestine (Zittel et al., 1998; Freeman et al., 1999; Glowka et al., 2015). The delay in gastric emptying and intestinal transit caused by endotoxin in mice and experimental peritonitis in rats is likewise mitigated by CGRP_8–37_ (Julia and Buéno, 1997; De Winter et al., 2009).

While CGRP released from extrinsic sensory neurons participates in gastrointestinal motor inhibition under particular pathological conditions, CGRP released from intrinsic enteric neurons plays a physiological role in initiating peristaltic (propulsive) motility in the intestine. This role is at first glance difficult to extract from a multitude of reports describing both excitatory (contractile) and inhibitory (relaxant) motor actions of CGRP in isolated segments or strips of the intestine. However, local effects of the peptide such as muscle relaxation (Holzer et al., 1989; Barthó et al., 1991; Barthó and Holzer, 1995; Sun and Benishin, 1995; Maggi et al., 1996; Rekik et al., 1997; Clifton et al., 2007), inhibition of acetylcholine release from myenteric neurons (Schwörer et al., 1991) or activation of inhibitory neural pathways (Holzer et al., 1989) reflect only stationary aspects of coordinated propulsive motility. Experiments with a more integrated view on intestinal propulsion demonstrate that CGRP stimulates motor patterns that are relevant to the aboral movement of the intestinal contents. Thus, CGRP induces phasic contractions of the circular muscle of the guinea-pig small intestine due to stimulation of cholinergic neurons (Holzer et al., 1989). Close intraarterial administration of CGRP to the canine ileum evokes rhythmic phasic contractions at smaller doses and giant migrating contractions at higher doses, effects that are prevented by CGRP_8–37_ (Sarna, 2003) although neither CGRP nor CGRP_8–37_ infused to dogs modifies the duration of the migrating motor complex during the interdigestive period (L’Heureux et al., 2000).

Detailed analysis of the stimulant effect of CGRP on intestinal propulsion has disclosed that CGRP-expressing IPANs in the rodent intestine play a crucial role in initiating those enteric reflexes (ascending contraction and descending relaxation of the circular muscle) that enable peristaltic movements to be triggered and to propagate in an aboral direction (Figure 2). In line with this implication, mechanical or chemical stimulation of the intestinal mucosa leads to release of CGRP from enteric sensory neurons (Grider, 1994; Foxx-Orenstein et al., 1996; Grider et al., 1998; Roza and Reeh, 2001). More specifically, when stimulated by mucosal stroking or 5-hydroxytryptamine (5-HT) released from enterochromaffin cells, CGRP-expressing IPANs lead to activation of ascending excitatory and descending inhibitory pathways in the enteric nerve plexus underlying peristalsis (Figure 2) in the rat, guinea-pig and mouse small and large intestine, this implication of CGRP being prevented by CGRP_8–37_ (Grider, 1994, 2003; Foxx-Orenstein et al., 1996; Grider et al., 1998; Pan and Gershon, 2000). When muscle stretch is used to stimulate the peristaltic motor pathways, CGRP released from capsaicin-sensitive extrinsic sensory neurons also comes into play (Grider, 1994). The prokinetic action of bile acids in the mouse colon is mediated by a similar mechanism, given that the effect of deoxycholic acid to stimulate peristalsis is inhibited by CGRP_8–37_ (Alemi et al., 2013). The stimulant effect of CGRP on peristalsis is consistent with the peptide’s action to depolarize enteric neurons and enhance their excitability (Palmer et al., 1986) and to potentiate excitatory neurotransmission to the circular muscle of the guinea-pig colon (Kojima and Shimo, 1995; Maggi et al., 1997).

### Functional Evidence for a Role of Calcitonin Gene-Related Peptide in Stimulating Intestinal Secretion and Causing Diarrhea

Motor activity in the gut is intimately related to intestinal ion and water secretion, given that an increase in the intraluminal volume of fluid and chyme will have a mechanical impact on the mucosa as well as gastrointestinal wall, in this way stimulate propulsive motility, and in combination with increased water secretion give rise to diarrhea. The intestinal mucosa is supplied by CGRP-positive intrinsic enteric as well as extrinsic sensory neurons which are strategically located to influence secretory processes. Thus, when these neurons are activated, epithelial ion transport in the mouse colon is enhanced by release of CGRP from submucosal enteric neurons (Tough et al., 2018). CGRP stimulates chloride secretion in the rat colonic mucosa by activating CGRP receptors that are preferentially located on the basolateral surface of epithelial cells (Cox, 1995). In contrast, CGRP’s secretory action in the guinea-pig colon is mediated by enteric neurons (McCulloch and Cooke, 1989). It may be added that the distension-evoked release of CGRP in the mouse colon is accompanied by a release of prostaglandin E_2_ (Roza and Reeh, 2001), which is known to play a role in intestinal secretion and diarrhea (Rask-Madsen et al., 1990). The prosecretory effect of CGRP is also seen *in vivo*, given that intravenous administration of the peptide enhances ion and water secretion into the rat colon (Rolston et al., 1989). In addition, evidence for a prosecretory role of CGRP comes from reports that inhibition of endogenous CGRP activity by knockdown of the CLR subunit of the CGRP receptor (Bhargava et al., 2013) or treatment with the receptor antagonist CGRP_8–37_ (Keates et al., 1998) attenuates intestinal water secretion induced by *Clostridioides* (previously termed *Clostridium*) *difficile* toxin A. In addition, the accelerated gastrointestinal transit evoked by ovalbumin anaphylaxis is blunted in mice deficient in RAMP1, the other subunit of the CGRP receptor (Yoshikawa et al., 2011).

The implications of CGRP in intestinal ion and water secretion are confirmed by the ability of exogenous CGRP to induce overt diarrhea in dogs and mice. Specifically, intravenous infusion of CGRP to dogs causes jejunal electrolyte and water secretion and causes diarrhea in the majority of animals (Reasbeck et al., 1988). Intraperitoneal administration of CGRP to mice likewise triggers diarrhea as assessed by stool consistency and weight, an effect that is reproduced by intracerebroventricular administration of the peptide which appears to cause diarrhea only following leakage to the periphery (Kaiser et al., 2017). Intraperitoneal pretreatment of the animals with CGRP antibodies is able to prevent the CGRP-induced diarrhea, while olcegepant is less efficacious in blunting the laxative action of the peptide (Kaiser et al., 2017).

Taken together, there is substantial evidence from studies in laboratory animals that CGRP can stimulate propulsive motor activity and secretion in the intestine, which leads to diarrhea. If these effects were physiologically relevant, CGRP antagonism/neutralization would be expected to delay gastrointestinal transit and bring about a functional state resembling constipation. Unfortunately, there is only limited information as to whether CGRP receptor antagonists or CGRP antibodies interfere *per se* with physiological gut function in laboratory animals. Thus, gastric corpus motility (Zittel et al., 1994), gastric emptying (De Winter et al., 2009), gastrointestinal transit (Glowka et al., 2015), and colonic transit (Zittel et al., 1998) in rats and mice remain unaltered by a single administration of CGRP_8–37_, olcegepant or a monoclonal CGRP antibody. One study, though, has shown that CGRP_8–37_ delays gastrointestinal transit in mice (De Winter et al., 2009). Notwithstanding these inconclusive observations, the CGRP-induced diarrhea in mice (Kaiser et al., 2017) and dogs (Reasbeck et al., 1988) attests to the peptide’s ability to stimulate intestinal transit and secretion, a contention that has been confirmed in humans (Falkenberg et al., 2020).

## Evidence for a Role of Calcitonin Gene-Related Peptide in Stimulating Intestinal Propulsion and Secretion in Humans

### Expression of Calcitonin Gene-Related Peptide and Calcitonin Gene-Related Peptide Receptors in the Human Intestine

Although the evidence gathered in experimental studies with laboratory animals attest to CGRP’s activity in enhancing intestinal peristalsis and secretion, species differences between laboratory animals and humans cannot *a priori* be neglected. Thus, it is important to review the available literature for information as to how CGRP impacts on intestinal motility and secretion in humans. As a matter of fact, however, the presence of CGRP and its physiological and pathophysiological implications in the human digestive tract have not as extensively been studied as in the gut of laboratory animals. The available information indicates that CGRP-like immunoreactivity in the human small intestine occurs not only in neuronal somata and fibers but also in mucosal endocrine cells (Timmermans et al., 1992; Brehmer et al., 2004; Cottrell et al., 2012). CGRP-positive enteric neurons are more abundant in the submucosal plexus than in the myenteric plexus, and some of the neuronal somata containing CGRP in the myenteric plexus of the human small intestine appear to be IPANs with a Dogiel type II morphology (Timmermans et al., 1992; Brehmer et al., 2004). Besides the peptide, CLR and RAMP1, the two subunits of functional CGRP receptors, have also been localized to the human stomach, small intestine and colon (Hagner et al., 2002; Cottrell et al., 2012). CLR-like immunoreactivity occurs in the myenteric and submucosal plexus, in nerve fibers within the circular and longitudinal muscle as well as in mucosal endocrine cells (Hagner et al., 2002; Cottrell et al., 2012). In addition, CLR and RAMP1 have been co-localized in the myenteric plexus where they are thought to form functional cell-surface receptors (Cottrell et al., 2012).

### Functional Evidence for a Role of Calcitonin Gene-Related Peptide in Stimulating Intestinal Motility and Secretion and Causing Diarrhea

Only a limited number of studies has addressed the actions of CGRP on motor activity and secretory processes in isolated preparations of the human intestine. CGRP has been reported to have a weak relaxant effect in muscle strips of the human ileum and colon exposed to electrical field stimulation (Maggi et al., 1990; Smith and Smid, 2005). The ability of CGRP to instigate peristaltic motor activity has been studied in segments of the human jejunum. As seen in the rodent gut (Figure 2), mucosal stroking, 5-HT released from enterochromaffin cells or exposure to a 5-HT_4_ receptor agonist causes the release of CGRP, presumably from IPANs, which gives rise to ascending contraction and descending relaxation of the circular muscle, these motor reflexes being prevented by CGRP_8–37_ (Foxx-Orenstein et al., 1996; Grider et al., 1998). It is also likely that CGRP can stimulate chloride secretion in the mucosa of the human intestine as deduced from the prosecretory effect of CGRP in two human epithelial cell lines (Cox and Tough, 1994).

In view of the potential of erenumab to cause constipation (occasionally of a severe nature), as seen already in some pre-approval RCTs, Falkenberg et al. (2020) examined the effect of a 2-h intravenous infusion of CGRP (1.5 μg/min) to 30 healthy volunteers on 2 different days. CGRP was found to induce symptoms of gastrointestinal hypermotility in 93% of the participants on both study days. Rumbling, stomach pain, nausea, an urge to defecate and diarrhea were the most commonly experienced adverse effects during the infusion of the peptide. The symptoms were first noted after a delay of 20 min, on average reached a peak after 60 min and, with the exception of nausea, disappeared shortly after the infusion had been stopped. Since the symptoms were not prevented by sumatriptan, Falkenberg et al. (2020) classified the CGRP-induced disturbances of gastrointestinal function as a direct effect of the peptide on the gut.

With regard to the potential of CGRP antagonism/neutralization to cause constipation it is also relevant to ask whether idiopathic chronic constipation (ICC) may involve a change in the gastrointestinal CGRP system. However, this question is not yet possible to answer. While the number of CGRP-immunoreactive nerve fibers in the colonic myenteric ganglia of ICC patients was found to be markedly enhanced in one study (Dolk et al., 1990) it remained grossly unaltered in another study (Sjölund et al., 1997).

## Diarrhea Associated with Neuroendocrine Tumors: Possible Involvement of Calcitonin Gene-Related Peptide

CGRP was discovered when it was realized that alternative processing (tissue-specific splicing) of the mRNA for calcitonin in the parafollicular (C) cells of the rat thyroid leads to production of CGRP, this peptide being widely expressed in neuronal tissue (Rosenfeld et al., 1983). Following the isolation of CGRP from patients with medullary thyroid carcinoma (MTC) (Morris et al., 1984), the peptide was, together with calcitonin, found to be expressed in the majority of MTCs and released into the circulation (Schifter et al., 1986; Takami et al., 1990; Hanna et al., 1997). Watery diarrhea has been reported to occur in some 30% of patients with MTC, this symptom being variously attributed to excess production of calcitonin and other factors (Hanna et al., 1997). CGRP doses administered to dogs mimicking the elevated plasma levels of CGRP found in human patients with MTC are able to stimulate water secretion in the small intestine and elicit diarrhea in 4 of 6 dogs (Reasbeck et al., 1988). The contention that high MTC-derived concentrations of CGRP in the circulation could contribute to the watery diarrhea accompanying MTC has likewise been put forward by Rolston et al. (1989) on the basis of the prosecretory effect of CGRP seen in rats. CGRP and calcitonin are also expressed in other neuroendocrine tumors associated with diarrhea such as carcinoid (Takami et al., 1990) and prostatic adenocarcinoma (Shulkes et al., 1991). The circulating levels of CGRP in prostatic adenocarcinoma correlate with the presence of diarrhea, and both the plasma levels of CGRP and the associated diarrhea have been found to be resistant to octreotide, a somatostatin analog commonly used in an attempt to mitigate tumor-associated symptoms (Shulkes et al., 1991).

Although no direct proof has yet been provided that MTC-associated diarrhea can be mitigated by CGRP antagonism or neutralization, the findings that elevated plasma levels of CGRP are associated with diarrhea indirectly support the contention that CGRP is able to stimulate intestinal motility and secretion. At the same time, this argument raises the possibility that CGRP antagonism could be beneficial in controlling the diarrhea associated with CGRP-producing tumors.

## Mechanistic Basis of Constipation Caused by Anti-Calcitonin Gene-Related Peptide Migraine Therapeutics

### Evidence Indicating That Constipation Results From Inhibition of Calcitonin Gene-Related Peptide’s Stimulant Effect on Intestinal Peristalsis and Secretion

**FIGURE 3:**
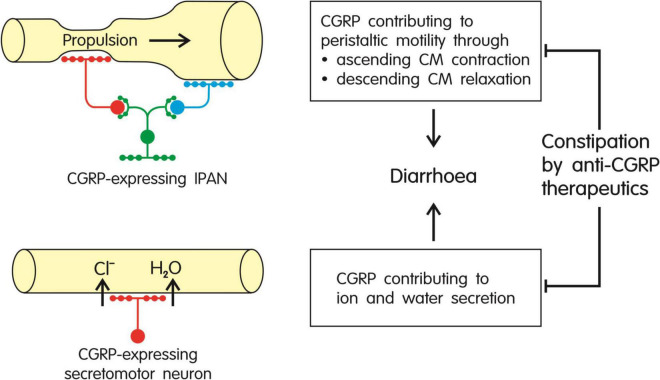
Summary of the major effects of CGRP in the gut: stimulation of peristaltic motor activity and increased secretion of ions (primarily Cl^–^) and water, both effects in combination causing diarrhea. As these effects of the peptide are likely to contribute to the maintenance of propulsive motility, ion and water homeostasis and intestinal transit, inhibition of CGRP signaling by anti-CGRP migraine therapeutics is expected to cause constipation. CM, circular muscle; IPAN, intrinsic primary afferent neuron.

The information reviewed herein attests CGRP both a motility-stimulating and prosecretory action in the intestine of various mammalian laboratory animals as well as humans (for a schematic summary see Figure 3). As there is evidence that CGRP is involved in the maintenance of peristaltic motor activity in the rodent and human gut, constipation evoked by CGRP antagonism/neutralization can be explained to result from interference with the physiological function of the peptide in the small and large intestine. In the gut, CGRP is expressed by distinct intrinsic enteric neurons and extrinsic primary afferent nerve fibers originating primarily in the dorsal root ganglia. The intrinsic enteric CGRP neurons participate in neuronal pathways that play a role in the initiation of propulsive motor activity and secretion of ions and water into the intestinal lumen (Figure 3). CGRP, in particular, is expressed in IPANs, intrinsic sensory neurons, of the mouse, rat, guinea-pig and pig (Brehmer et al., 2002; Furness et al., 2004; Wolf et al., 2007; Mitsui, 2009, 2011; Hibberd et al., 2018; Smolilo et al., 2020) and has also been localized to IPANs of the human small intestine (Timmermans et al., 1992; Brehmer et al., 2004). The CGRP-expressing IPANs contribute to the effect of mucosal stimulation to initiate peristaltic movements, given that both the ensuing ascending contraction and descending relaxation of the circular muscle in the mouse, rat, guinea-pig and human intestine *in vitro* are blunted by the CGRP receptor antagonist CGRP_8–37_ (Foxx-Orenstein et al., 1996; Grider et al., 1998; Pan and Gershon, 2000; Grider, 2003).

Coordinated peristaltic motility requires a consecutive interplay of (ascending) contraction and (descending) relaxation of the circular muscle so that the contraction can propel the intestinal content aborally to an adjacent segment that is relaxed. The implication of CGRP in this motor pattern may explain why both excitatory (contractile) and inhibitory (relaxant) responses to CGRP have been reported in various isolated preparations of the intestine. It should not go unnoticed in this respect that experimental studies have provided consistent evidence that exogenous CGRP slows gastric emptying in rats (Lenz, 1988; Plourde et al., 1993; Julia and Buéno, 1997) and dogs (L’Heureux et al., 2000). Whether the peptide has a similar action in humans has not yet been examined. If CGRP were to inhibit gastric emptying in humans, this action might be a factor contributing to nausea which is another documented adverse effect of anti-CGRP migraine therapeutics. It is worth noting in this context that an ongoing phase 4 clinical trial investigates in which way treatment of adult migraine patients with erenumab or galcanezumab affects gastric emptying and gastrointestinal transit (ClinicalTrials.gov identifier: NCT04294147)^1^.

The important clues to CGRP’s role in facilitating intestinal propulsion and ion/water secretion obtained in *in vitro* investigations have been confirmed by the ability of CGRP to induce diarrhea *in vivo* as observed in mice (Kaiser et al., 2017), dogs (Reasbeck et al., 1988), and humans (Falkenberg et al., 2020). Thus, the pharmacological and physiological activities of CGRP in the intestine of laboratory animals can be extrapolated to humans. Although pretreatment of mice with CGRP antibodies and olcegepant is able to blunt CGRP-induced diarrhea (Kaiser et al., 2017), there is scarce information as to whether CGRP antagonism/neutralization in laboratory animals has *per se* an adverse effect on intestinal propulsion *in vivo*. While one study found CGRP_8–37_ to delay gastrointestinal transit in mice (De Winter et al., 2009), most studies failed to see gastric corpus motility (Zittel et al., 1994), gastric emptying (De Winter et al., 2009), gastrointestinal transit (Glowka et al., 2015) and colonic transit (Zittel et al., 1998; Glowka et al., 2015) in rats and mice to be altered by CGRP_8–37_, olcegepant or a CGRP antibody. In classifying these findings two aspects need be taken into account. On the one hand, peptidic antagonists such as CGRP_8–37_ appear to have insufficient access to endosomal compartments that are also relevant to CLR signaling (Yarwood et al., 2017). On the other hand, in laboratory animals CGRP antibodies and antagonists have been tested only in short-term experiments whereas in humans CGRP-directed migraine therapeutics are used repeatedly for a prolonged period of time.

On the basis of the available evidence it can be concluded that CGRP plays a physiological role in stimulating intestinal propulsion, secretion and transit in humans. Blockade of this function is poised to lead to constipation as shown by the adverse effect of anti-CGRP therapeutics, a finding which at the same time confirms the involvement of CGRP in the maintenance of intestinal transit. In view of this reasoning, constipation cannot be classified as an indirect effect of CGRP-directed migraine therapeutics, as hoped by Haanes et al. (2020), but need be regarded as the result of blockade of CGRP signaling in the neural pathways underlying intestinal motor activity and secretion. In its medical implication this contention poses a major pharmacological challenge: How can the adverse effect of anti-CGRP therapeutics in the intestine be kept in check without compromising their anti-migraine action which is also thought to take place at a peripheral site outside the blood-brain barrier (Raffaelli and Reuter, 2018; Hargreaves and Olesen, 2019; Johnson et al., 2019; Messlinger and Russo, 2019; Christensen et al., 2020; Noseda et al., 2020)? At any rate, the risk of constipation associated with the use of antibodies targeting CGRP or its receptor need be accounted for in therapeutic decision making and treatment monitoring (Deligianni et al., 2021).

### Open Questions and Future Challenges

In its quest to explain whether constipation caused by anti-CGRP therapeutics can be explained by antagonism of the physiological role of CGRP in the intestine, this review was based on the disparity between the comparably low rates of constipation seen in the pre-approval RCTs of antibodies targeting CGRP or its receptor and the much higher rates reported in post-approval real-world surveys. Constipation was noted only in one RCT of atogepant (Ailani et al., 2021) and fremanezumab (Ferrari et al., 2019) but in three phase 3 trials of erenumab (Goadsby et al., 2017; Tepper et al., 2017; Takeshima et al., 2021) although in five other RCTs of erenumab (Sun et al., 2016; Dodick et al., 2018a; Reuter et al., 2018; Goadsby et al., 2020; Hirata et al., 2021) constipation went unmentioned. It was unforeseen, therefore, that substantial rates of constipation in patients treated with erenumab, fremanezumab and galcanezumab (see Table 1) were reported in post-approval surveys. This outcome contradicted the initial contention that the adverse effect profile of anti-CGRP migraine therapeutics is favorable and within the limits expected for this class of therapeutics. However, concerns that gastrointestinal adverse effects, particularly constipation, may be underrated and underreported were voiced before the post-approval survey data went public (Hargreaves and Olesen, 2019; Robbins, 2019; Falkenberg et al., 2020).

A straightforward explanation for the significantly higher constipation rates in migraine patients on a CGRP-targeting therapy under real world conditions as compared to RCT conditions is not yet available. Differences in the patient populations in terms of medical history and comorbidities as well as a more detailed assessment of adverse events and disturbances of life quality are likely to be contributory factors (Lambru et al., 2020; Faust et al., 2021). Important information may also be deduced from a future comparison of the real world constipation rates associated with the long-term use of gepants, on the one hand, and antibodies targeting the CGRP system, on the other hand. Differences in the constipation rates may provide clues as to whether pharmacodynamic and/or pharmacokinetic factors play a role. The two classes of anti-CGRP therapeutics differ profoundly in their pharmacokinetic profile, given that gepants have a comparatively short biological half-life (5–11 h) while the CGRP and CGRP receptor antibodies have a biological half-life of 27–31 d (for reviews see Szkutnik-Fiedler, 2020; Omaer et al., 2021). Thus it may make a difference if CGRP signaling in the intestine is continuously blocked by antibody therapeutics for a prolonged period of time as compared to a more intermittent receptor blockade caused by gepant therapy.

In addition, there may also be appreciable pharmacodynamic differences between the different classes of anti-CGRP migraine therapeutics. The CGRP receptors operating in the human intestine that are blocked by the gepant and antibody therapeutics have not yet been characterized in sufficient detail. Together with calcitonin, CGRP, amylin, adrenomedullin and intermedin form a family of peptides that can use different receptors of a related structure for their biological actions (Hay et al., 2018). CGRP is not only an agonist at the CGRP receptor (a CLR/RAMP1 heterodimer) but also at the AMY_1_ receptor, a heterodimer of CTR (calcitonin receptor) and RAMP1, at which amylin is also an agonist (Hay et al., 2018). It has recently been reported that the available CGRP-directed migraine therapeutics differ in their receptor selectivity and their signaling via cAMP production (Bhakta et al., 2021). While erenumab and telcagepant antagonize not only CGRP but also adrenomedullin and intermedin at cAMP signaling through the CGRP receptor, fremanezumab is a specific antagonist of CGRP at the CGRP receptor (Bhakta et al., 2021). In contrast, erenumab and telcagepant also bind to the AMY_1_ receptor and inhibit cAMP signaling through this receptor (Bhakta et al., 2021). If different receptors such a CLR/RAMP1 and CTR/RAMP1 were to be involved in the action of CGRP and related peptides on intestinal motor activity and secretion, different classes of anti-CGRP therapeutics may also differ in their propensity to cause constipation. Indeed, the potency of CGRP_8–37_ in antagonizing the prosecretory response to α-CGRP in the rat and mouse colon and in the human epithelial cell line Colony-29 is very low as compared with other effects of the peptide (Cox and Tough, 1994; Cox, 1995; Esfandyari et al., 2000; Tough et al., 2018), which has been taken to conclude that a different type of receptor mediates the CGRP_8–37_-insensitive action of CGRP.

An important open question is: what medications are available to control the constipation brought about by anti-CGRP therapeutics while their anti-migraine action is maintained? Arguably this goal may be best achieved by prokinetic and laxative drugs that are suitable for long-term use and have a site of action that is restricted to the gut. In this respect it is reassuring to note that the repository of prescription medications used to treat chronic constipation has been appreciably expanded in the past 15 years (Herbert and Holzer, 2008; Bassotti et al., 2021; Wechsler and Shah, 2021). The drugs at hand include 5-HT_4_ receptor agonists (e.g., tegaserod, prucalopride), osmotic laxatives (e.g., polyethylene glycols), stimulant laxatives (e.g., bisacodyl) and a number of compounds that act on newly identified gastrointestinal targets. These include lubiprostone (an activator of CIC2 chloride channels), linaclotide and plecanatide (activators of guanylate cyclase C), tenapanor (an inhibitor of the sodium-hydrogen exchanger NHE3) and elobixibat (an inhibitor of the ileal bile acid transporter). It is not possible to judge which of these medications would be particularly suitable for the control of constipation caused by CGRP blockade before any experience based on clinical studies has been gained. Acetylcholinesterase inhibitors such as neostigmine and distigmine which enhance muscle tone are used to treat gastrointestinal pseudo-obstruction disorder but not chronic constipation because they induce muscle spasms at higher dosage, inhibit rather than promote peristalsis (Fruhwald et al., 2004), affect other peripheral cholinergic systems (e.g., in urinary system, heart and lung) and may lead to cholinergic crisis. The gastroprokinetic drugs domperidone (a dopamine D_2_ receptor antagonist) and metoclopramide (a dopamine D_2_ and 5-HT_3_ receptor antagonist and 5-HT_4_ receptor agonist) are likewise contra-indicated since safety concerns led EMA to restrict these drugs to short-term use.

In a final note, the activity of antibodies targeting the CGRP system to bring about constipation may be exploited therapeutically in intestinal disorders in which endogenous CGRP contributes to diarrhea, as has been suggested by Kaiser et al. (2017). For instance, it would be an option to explore the capacity of anti-CGRP therapeutics to control the diarrhea associated with neuroendocrine tumors such MTC, carcinoid and prostatic adenocarcinoma, in which the overexpression of CGRP results in elevated circulating levels of the peptide (Morris et al., 1984; Schifter et al., 1986; Takami et al., 1990; Shulkes et al., 1991; Hanna et al., 1997). If CGRP were a causative factor, CGRP blockade could be another approach to control diarrhea, complementing established treatment modes including somatostatin analogs such as octreotide. Other pathological conditions in which anti-CGRP therapeutics could have a beneficial effect include diarrhea caused by bacterial infection, given that the intestinal water secretion and inflammation evoked by *Clostridioides difficile* toxin A in rats depends on endogenous CGRP (Keates et al., 1998; Bhargava et al., 2013). Similarly, the accelerated gastrointestinal transit evoked by ovalbumin anaphylaxis, which is blunted in mice deficient in RAMP1 (Yoshikawa et al., 2011), suggests that food allergy-associated diarrhea could be susceptible to the therapeutic use of CGRP signaling blockers. Inflammatory bowel disease and the diarrhea-predominant form of irritable bowel syndrome (IBS-D) are also pathologies characterized by enhanced intestinal secretion and transit. It is unlikely, however, that inhibition of CGRP signaling is a therapeutic option under inflammatory conditions, given that both exogenous and endogenous CGRP protects from experimentally induced colitis in laboratory animals (Evangelista and Tramontana, 1993; Reinshagen et al., 1998; Mazelin et al., 1999; Clifton et al., 2007; Thompson et al., 2008; Engel et al., 2011; de Jong et al., 2015; Kawashima-Takeda et al., 2017; Yamasaki et al., 2020). In conclusion, the capacity of antibodies targeting CGRP or its receptor to inhibit intestinal secretion and transit offers a new lead for therapeutic exploitation in select pathologies in which CGRP overactivity is a pathophysiological factor.

## Author Contributions

PH and UH-P wrote the manuscript and drew the figures. Both authors contributed to the article and approved the submitted version.

## Conflict of Interest

The authors declare that the research was conducted in the absence of any commercial or financial relationships that could be construed as a potential conflict of interest.

## Publisher’s Note

All claims expressed in this article are solely those of the authors and do not necessarily represent those of their affiliated organizations, or those of the publisher, the editors and the reviewers. Any product that may be evaluated in this article, or claim that may be made by its manufacturer, is not guaranteed or endorsed by the publisher.

## Footnotes

1
https://clinicaltrials.gov/ct2/show/NCT04294147
